# Exploring Children’s Creative Self-Efficacy Affected by After-School Program and Parent–Child Relationships

**DOI:** 10.3389/fpsyg.2020.02237

**Published:** 2020-09-15

**Authors:** Chen-Chu Liang, Yu-Hsi Yuan

**Affiliations:** ^1^Department of Education, National University of Tainan, Tainan, Taiwan; ^2^College of Economics and Management, Zhejiang Normal University, Jinhua, China

**Keywords:** parenting style, after-school program, parent–child relationships, creative self-efficacy, learning environment

## Abstract

This study aimed to verify the relationship among children’s creative self-efficacy, parenting style, parent–child relationship, and after-school program. Judgmental sampling was used for subject selection from Taiwan. There are 550 valid participants composed of elementary school to junior high school students; their data were put into the statistical process. The multiple regression analysis was applied in this study. The survey tool was developed based on literature review and related articles. Research result supported the idea that the after-school program was the most significant variable that affected the student’s creative self-efficacy. The “punitive discipline” and “autonomy support” of parenting style can affect positive parent–child relationships as well as students’ creative self-efficacy. Evidence supported the notion that “negative parent–child relationships” will not motivate students’ creative self-efficacy. Besides, the after-school program plays an important role in the students’ creative self-efficacy independently.

## Introduction

The development of a child’s creativity must take root in his or her experience in life. A supportive family and a better parent–child relationship will certainly contribute to a better climate for creativity (Zhang et al., 2018). Growing up in such an environment, a child is expected to be inspired to be creative from an early age (Lim and Smith, 2008; Wu et al., 2018). The child will grow up to be independent and capable of solving problems independently and demonstrate creativity even when he or she is still very young (Kwaśniewska et al., 2018). Researchers such as Chong et al. (2019) believe that the development of creativity is mainly due to heredity and environment (P. 5). Most educators hold the same view as the Italian educator Montessori in believing that all the space layout, equipment, items, etc. in the environment prepared by the parents will have a pivotal impact on a child’s development as long as it is a right environment (Denervaud et al., 2019; Fleming et al., 2019). Obviously, the right environment is very important to children’s growth. For students of primary and middle schools, aside from family and schools, other environments such as after-school classes or tutoring classes are also critical (Manichander, 2016; Dai and Xu, 2019). So far, the family and school environments with great impact on children’s development have extensively been studied and discussed, but not the after-school programs. This study is motivated by the lack of such studies.

In addition, parenting issues are always the focus of attention drawn from many scholars in the fields of education, sociology, psychology, and social work. In general, “parenting” refers to the process in which parents teach their children, including parents’ beliefs how children should be raised, as well as what are the right concepts, disciplines, and behaviors they expect from their children (McCarthy et al., 2017; Becker et al., 2019; Bell, 2020). Through careful nurturing and interactive relationships, parenting has a profound impact on personal development (Moltafet et al., 2018). Parents’ attitudes toward nurturing and their behaviors in the process will, therefore, be closely related to the development of their children’s mental state, personality, emotions, adjustability to social life, and learning achievements when they grow up (van der Pol et al., 2016; McCarthy et al., 2017; Jagers et al., 2019; Bell, 2020). Because parents’ concept of parenting would influence their parenting behaviors, the whole process of parenting will certainly have a great impact on children’s development (Moltafet et al., 2018). This is especially true in today’s situation in Taiwan, where parents are mostly busy with their work or career, under the pressure of keeping up with rapid economic development, and thus have little time left to be with their children and take care of them. The formation of such a communication gap between parents and their children leads to the second motivation of this study’s concern about parenting issues.

On the other hand, our society has witnessed the rise of after-school care in the context of the structural change in most families, which has been driven by the economic needs and the improvement of women’s status in the family as well as their working ability. As a result, women’s employment opportunities have increased and their functions in the family are different (Malm et al., 2017; Smith et al., 2018). Related research also found that school-age children have much higher needs in care than pre-school children. This is so because most employed mothers prefer to continue their work and those mothers who left their jobs during pregnancy would also like to rejoin the workforce after their children have attended primary school. As a result, the need for child care after school is urgently needed (Naito and Wie, 2018). In a busy modern society, most parents in Taiwan have more than one job to make ends meet. Their busy work schedule would not allow them to pick up their children in a time when school is over. This is why after-school tutoring classes are in great demand (Chen H. H. et al., 2016; Pai et al., 2018). The after-school tutoring classes not only are entrusted to take care of students when the parents are unable to pick them up when school is over but also provide an important incubation environment for students’ coursework and personality development. How the curriculum of after-school tutoring classes would impact students’ development has thus become the third motivation of this study.

## Literature Review

### Creative Self-Efficacy

In the inaugural speech delivered by Guilford in 1950, when he was the chairman of the American Psychological Association (APA), he actively promoted the importance of creativity. At that time, Guilford regarded creativity as a personal characteristic. Later, Rhodes (1961), after having analyzed the definitions of creativity given by nearly 50 scholars, proposed “the four Ps” factors of creativity and pointed out that creativity is composed of the factors person, process, pressure/place, and product. This is a view widely accepted by scholars today. Mednick (1962) and Torrance (1966) view creativity from a different perspective of history and advocate that creativity is formed by a series of thinking processes. Wallach and Kogan (1965) believe that creativity is the evaluation of products or achievements. Scholars in education tend to interpret creativity from the perspective of interaction (Sternberg, 1988; Gardner, 1999; Sternberg et al., 2004). Creativity is generally defined as the generation of ideas or products that are both novel and appropriate such as correct, useful, valuable, or meaningful (Amabile et al., 1996; Amabile, 2012). Products are considered to be creative based on the appraisers’ consensus on something special displayed by the products. They believe that if the observer agrees that a certain product or a certain response is creative, then the product is deemed creative. In addition, Sternberg and Lubart (1995) view creativity from an investment perspective. They believe that creativity illustrates the use of a person’s six resources of creativity, namely, intelligence, knowledge, intellectual styles, personality, motivation, and environment, to buy low and sell high in the market of creative products. Although scholars in different research fields believe in different definitions of creativity, most scholars generally agree on the definition of creativity proposed by Stein (1974); that is, creativity is a process that creates a novel work that is accepted as tenable or useful or satisfying by a group at some point in time. Among the features mentioned in his definition of creativity, novelty, tenability, usefulness, and group satisfaction of and for the products are those agreed upon by most scholars today.

The study of Amabile (1983), who adopted measuring methodologies from other researchers, focuses on the measurement of creative products. She believes that creative products must meet two criteria, that is, novelty and appropriateness. Because a product of novelty is not creative enough, it has to be appropriate in the sense that it also fulfills its purpose as a product, to be useful and satisfactory. In other words, both features are equally important for a product to be creative. Creativity is the creative thinking demonstrated in the behavior and ideation of the production process or the product. From the cognitive point of view, it is demonstrated in sensitivity to problems as well as fluency, flexibility, originality, and elaboration in thinking (Guilford, 1967; Hu et al., 2016; Singh and Kumar, 2017). Sensitivity refers to the ability to perceive the problems among objects with sensitivity. It can discover relationships among them, or their deficiencies, unusualness, and supplements. This is the key ability of creativity. Fluency refers to the ability to come up with points or ideas in a short time. Flexibility refers to the ability to break through traditional thinking and be ready to make changes by looking at a problem from a different point of view. The ability to infer based on analogy is another kind of flexibility. Originality refers to the ability to come up with ideas that are different from others’ or to have a unique or unexpected response. Elaboration refers to the ability to add new elements or dimensions to the original concept or to keep on refining the old thoughts. With regard to the nature and content of creativity, scholars in the past have put forward different views, theories, and methods of measurement.

Lately, the new trend of creativity research tends to regard creativity as a multi-dimensional concept. In other words, the level of creativity is the convergence of forces driven by personal attentiveness, creation process, end products, and the environment. From the investigation of personal attention toward objects to the studies of the cultural and socio-ecological elements, the creativity can now be approached from diversified perspectives (Hsiao, 2011). Yeh et al. (2000) adopts a synthesis view to define creativity as “the process in which an individual in a specific domain produces an appropriate product with originality and added value.” This creative process involves the integration of cognition, affection, and skills and their effective application. This creative self-efficacy is the result of the interaction between the environment and the individual’s knowledge as well as his or her experience, intentions (including attitudes, tendencies, and motivations), skills, or strategies. It also involves a profound influence on creative self-efficacy.

The term “creative self-efficacy” is derived from the term and theoretical context of self-efficacy that has been introduced and defined by Albert Bandura (Bandura, 1977), who also defined it and looked into its theoretical background. Bandura (1977) defined self-efficacy as “a person’s estimate that a given behavior will lead to certain outcomes” (p. 193). Brockhus et al. (2014) defined creative self-efficacy as: “The belief the person has in his own ability to produce the creative outcome in a specific setting or in general” (p. 438).

Bandura (1997) believes that self-efficacy is an important condition for creativity, because self-efficacy will enhance motivation, and motivation will give priority to their thoughts and behaviors, and put more emphasis on their thoughts and behaviors. Thus, personal motivation will in turn trigger two factors: (1) the individual’s choice of a certain behavior and (2) the individual’s pursuit of this behavior. Besides, there are other concepts with similar senses, such as self-image, self-esteem, and self-confidence. Brockhus et al. (2014) believe that these concepts are related to an individual’s overall self-image. However, creative self-efficacy is different because it is not the same as the overall self-efficacy in that the individual’s creativity is related to specific self-efficacy. Overall self-efficacy is a kind of overall belief, and individual self-efficacy refers to an individual’s belief in his or her creative ability, which gives the individual the ability to successfully cope with the requirements of different tasks across domains. Based on the above discussions, this study holds on to the arguments of Brockhus et al. (2014) and bases its development of theory and the creation of questionnaires on the concept of creative self-efficacy.

### After-School Program

After-school programs are now used to call the after-school care used in the past. Lee (2001) pointed out that the names used by nursery practitioners include after-school nursery, after-school care, after-school care agents, after-school caretakers, etc. In 2006, the Ministry of Education revised the “Tutorial and Continuing Education Law,” declaring that from now on only the name “After-school Care Classes” will be used for “after-school care classes” held by elementary schools and “after-school nursery” under social administration divisions as well as private “after-school care centers.” Ljusberg (2018) believes that entrusted childcare services refer to services to supplement parental care, help with children’s upbringing when parents are absent, and provide organized care in diverse forms outside the family for a certain period of time. Even though some children have sometimes been placed under the care of some childcare providers, the parents still retain the main responsibility for raising their children, and the family is still the focus of children’s lives. Childcare services must be authorized by parents to shoulder the task of childcare when parents cannot take care of the kids themselves. The definition of the care for school-age children in the literature is generally based on daycare programs provided for children between 6 and 12 before or after the regular school time, and during winter and summer holidays when parents are unable to take care of their children themselves. Such services can be called after-school care for school-age children (Hjalmarsson, 2018). As such, after-school care aims at taking care of elementary school-age children. It is the kind of care that complements parenting functions between the time when school children are out of school and the time when the parents go home from work. They are required to help with children’s development and meet their needs during the growing stage and to provide parental care lest family functions should be lost, which can be expected to incur much more costs to the society in the future just to remedy the unwanted losses.

For school-age children, after-school care can effectively plan their curriculum, teaching, and counseling designed to inspire students to make use of their instinct to do active learning. In addition to cultivating their interest, children are also expected to develop different aspects of living skills. After-school care is useful for children of disadvantaged families. The “positive differential treatments” such as rehabilitation, stimulation, reinforcement, and so on are usually included in the objectives of after-school programs to promote equal educational opportunities. Thus, the purpose of after-school care lies in (1) making the family care more complete so that mothers would no longer worry too much about children’s lives and feel at ease at work; (2) providing children with a safe and a learning environment of frequent interactions, so that children can have healthy physical, mental, and personality development; (3) government, community, and the industry work in cooperation to promote to children’s total welfare through services of care, protection, education, together with counseling; (4) enhancing educational opportunities and quality for disadvantaged children, narrowing the gaps in children’s education, and upholding the values of justice and fairness in the society; (5) using after-school care service network to provide help to assist in raising children for families so that the community can work seamlessly with families (Plantenga and Remery, 2017). However, if we expect after-school programs, whether they are after-school childcare or after-school tutoring, to have a substantive effect on students’ development, how their curricula and functions would work remains to be thoroughly discussed and analyzed. This is sure to be an important research topic to investigate.

### Parenting Style

The degree of parents’ reaction toward their kids’ behavior, whether it is acceptance, rejection, or contradiction, and the parenting behaviors in their interaction with the child will help shape a child’s mode of mental reaction. When the child becomes a parent, the same mental reaction mode will then become an important factor of his or her response toward kids’ needs (Ensink et al., 2017). In addition to their roles as caregivers, regulators, and socialization facilitators, parents have a more important role as a good listener or a counselor to help their children go through emotional turmoil (Lieberman et al., 2018). Although most of the theoretical and empirical research in the past decades has mainly been focused on the various concepts of parenting, there is still a lack of consensus about the key elements and evaluation of parenting styles (Reid et al., 2015). Most of the available literature is based on Baumrind’s (1966, 1967, 1971) study of parenting styles, and many more studies use two simplified factors of emotional warmth and behavioral control to classify the styles into four categories. However, according to Baumrind’s (1966) original monographs, the variables in parenting are extremely complicated, which even include elements such as democracy and autonomy granting. In addition, the study of children’s puberty has found that parents are more and more concerned about different dimensions of psychological control, which has nothing to do with the factors that Baumrind’s traditional views focus on. According to the results of relevant research, it is relatively more important to examine psychological control factors during the parenting of pre-adolescent children (Aunola and Nurmi, 2004, 2005; Caron et al., 2006; Kuppens et al., 2009). It is true that some people argue that some important influential factors may be missed when the classification of parental control into a variety of different upbringing models is not adequately defined. It is therefore essential to have the key elements of parenting well defined so that the impact of different parenting styles can be assessed accordingly (Barber, 1996; Skinner et al., 2005).

It is also true that some researchers believe that a set of predetermined aspects of parental control prescribed by the current academic research of parenting might not be able to fully describe the actual dynamics of child upbringing. There should be other factors worthy of further exploration and discussion (O’Connor, 2002; Skinner et al., 2005). However, we find from the in-depth inductive analysis of the literature that previous studies have already proposed many influential factors for the raising of children, including democracy, involvement, discipline, monitoring, corporal punishment, contingent discipline, and inconsistent conflicts, which have given us a fairly complete set of variables in parenting (Gershoff, 2002; Locke and Prinz, 2002).

Moreover, the review of literature also suggests to us a lot of variables and parenting behaviors useful in helping parents to better kids’ emotional control and their autonomy support, such as anxious intrusiveness, power assertion, love withdrawal, children’s behavioral, emotional, and cognitive psychological control, conditional regard, hostility, child’s dependency-oriented behaviors, achievement-oriented psychological control, overprotection and overindulgence, scaffolding, responsiveness, etc., as well as elements like invalidation, guilt induction, excessive expectations, ridiculing, embarrassing in public, comparing to others, ignoring, and violation of privacy (Landry et al., 2002; Soenens et al., 2010; Barber et al., 2012). Then, a comprehensive classification can place them under three different types of parental control, namely, positive emotional warmth, autonomy support, and permissive discipline. Also included are negative punitive discipline and anxious intrusiveness (Reid et al., 2015).

In order to classify and differentiate parenting styles, this study adopts the views of Reid et al. (2015), constructing the parenting styles based on the following four factors – emotional warmth, punitive discipline, tolerant indulgence, and autonomy support – to lay the foundation of our research tools.

### Parent–Child Relationships

“Parent–child relationship,” as the name suggests, is the relationship between parents and children and a kind of interpersonal relationship formed by the interaction of both parties. Blake (2017) believes that the parent–child relationship belongs to the family ties, a kind of interpersonal relationship formed by the interaction between parents and children. Scharp and Thomas (2016) explained the parent–child relationship by referring to the attitude of parents toward the upbringing of their children and the psychological interaction between them and their parents. However, the interaction between people is a two-way street, and the behaviors and attitudes caused during the process will be different according to the reaction of the other party. That is to say, the behavioral interaction between the two parties will produce psychological interactions and affect each other. The parent–child relationship is a mutual relationship between parents influencing children and children influencing parents. Some studies in Taiwan hold the same view and believe that the parent–child relationship is the attitude and way of mutual treatment between parents and children. Through the process of interaction, they have the result of mutual influence. On the one hand, the attitude and behavior of parents toward children will affect their children. On the other hand, children’s behavior will also affect their parents’ attitudes and methods of discipline (Chen and Gau, 2016; Chen W. W. et al., 2016).

Generally speaking, the attitude or psychological contact between parents and children in the parent–child relationship is mainly about family life, and it is manifested in several specific situations, such as mutual conversation and communication, family atmosphere, handling of house chores, parental expectations, learning environment, and principles and methods concerning reward and punishment (Zimmer-Gembeck et al., 2019). Therefore, whether the parent–child interaction is good or bad can usually be seen from how the parent–child relationship in the family living situation is adjusted. García-López et al. (2016) define the adjustment of the parent–child relationship as referring to the aspects of mutual love–hate, rejection–acceptance, domination–autonomy, and restraint–indulgence between parents and children in the context of family life in terms of emotional, authoritative, and structural interactions in isotropic space. In addition, a good parent–child relationship depends on the proper upbringing of the children by the parents, adequate love exchanges between the parents and the children, and good communication between them. That is, the three dimensions of parent–child mutual care, love, and communication between parents and their children can be used as indicators of the parent–child relationship (Bergström et al., 2019; Scott et al., 2019). According to the comprehensive literature, it is obvious that parent–child relationships and their interactions have a pivotal impact on children’s growth and the shaping of their personality. However, the parent–child relationship between students receiving after-school tutoring and their parents as well as how their learning and creative development will be affected still begs for further study and clarification.

### Summary

From the comprehensive review of the literature, it is believed that the cultivation of students’ creativity mainly depends on their creative self-efficacy. However, in today’s social background and context, taking into consideration the fact that both parents are working and the subsequent problems of child upbringing, the after-school tutoring is now playing a key role in helping with students’ learning performance and inspiring their creativity potential (Nieting, 1983; Lee, 2001; Plantenga and Remery, 2017; Hjalmarsson, 2018; Ljusberg, 2018). If we consider the influence of the students ‘original family again, the parental style and parent–child relationship are the key to it, and it is also the underlying foundation for students’ development of personality and behavior patterns (Locke and Prinz, 2002; Kuppens et al., 2009; Scharp and Thomas, 2016; Ensink et al., 2017; Lieberman et al., 2018). Because the duration of after-school tutoring is synchronized with schools’ semester plan, its function is becoming important. It, therefore, has the attention of the education authorities both at home and abroad. On the other hand, the arrangement of the learning content for after-school tutoring has a great impact on students. In particular, there is not much relevant literature on how creative teaching would inspire students’ creativity. Under the nation’s education policy to inspire students to develop their creative abilities, schools in our education system are working hard to include courses that would benefit students’ development of their creativity. The after-school programs outside the school system are also trying their best to keep up with the same policy. However, the actual effect of after-school tutoring remains to be seen due to the fact that the literature of relevant research is still lacking. This is what this study wants to find out.

### Framework

**FIGURE 1:**
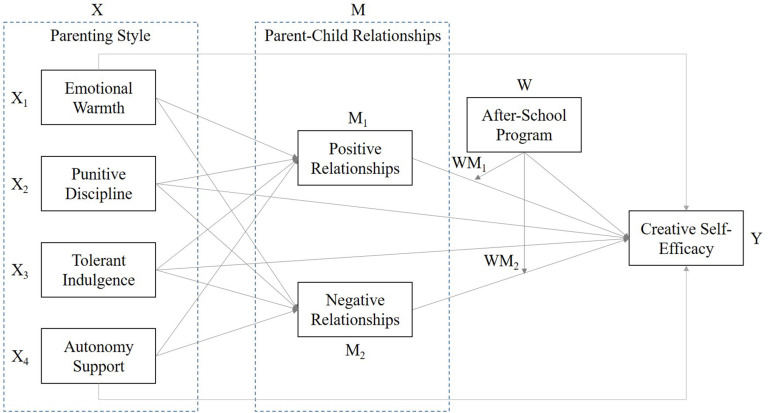
The framework of this study.

The framework of this research, as shown in Figure 1, followed the second stage moderation model and highlights the influence of parenting style on parent–child relationships, as well as how parent–child relationships and after-school programs would impact students’ creative self-efficacy. There are four facets of parenting style as independent variable (X), namely, Emotional Warmth, Punitive Discipline, Anxious Intrusiveness, and Autonomy Support, whereas parent–child relationship as mediator (M) reveals two facets of positive and negative relationships. In addition, the students’ creative self-efficacy is a dependent variable (Y) and the after-school program is a moderator (W) in the research framework. To estimate the moderating effect, it will calculate the W to Y and WX to Y (Hayes, 2013, 2018).

### Participants

The sampling in this study is mainly drawn from liberal arts tutoring classes and talent tutoring classes in Taiwan. This is beneficial for the researchers in this study because they work as the administrators of the two schools of liberal arts and talent programs. The general liberal arts institute mainly recruits students who are elementary school or junior high school students. The main courses listed are Chinese Language, Gifted Mathematics, Natural Science, English, and Abacus. The curriculum design is based on multiple learning tracks and enhanced after-school tutoring. The institute is noted for unit-based teaching courses of each subject, which includes the courses of innovative writing in Chinese, mental map learning, and oral skills. Gifted Mathematics integrates traditional cultural abacus and mental arithmetic teaching into Math to improve students through hand-on practices, which aim to enhance students’ computing ability and sense of speeding calculation, thus further developing students’ logical thinking. Natural Science emphasizes the combination of scientific experiments and hands-on operation. English emphasizes oral skills, grammar, and the use of lively activities to uphold students’ learning motivation. Through intentional sampling, students from after-school tutoring classes K1–K19 all over Taiwan who have expressed their willingness are invited to participate in the research survey by filling out the questionnaire. The survey participants are mainly students who have enrolled in the related after-school tutoring courses for the past year. A total of 600 questionnaires are sent out and 550 valid questionnaires are returned.

### Questionnaire Development

#### Item Generation

The research questionnaire is designed based on the relevant theories, research reports, and literature in the past, with the reference of some questionnaires appended to published academic papers (Liao, 2015). Back translation is done and reviewed to make sure that the questions are not in violation with the original in terms of meaning and they are not beyond the comprehension of primary and middle school students. Thus, the initial questionnaires are compiled. Table 1 shows the items and dimensions included in the questionnaire (La Heij et al., 1996; Gandek and Ware, 1998; Andersen et al., 2014).

**TABLE 1:** The items and dimensions of the developed questionnaire.

Factors	Dimensions	Items	Original source
Parenting style	Emotional warmth	4	Reid et al., 2015
	Punitive discipline	4	
	Anxious intrusiveness	4	
	Autonomy and support	4	
Parent–child	Positive relationships	5	Lo (2001)
Relationships	Negative relationships	4	
After-school program	–	6	Self-development
Creative self-efficacy	–	12	Brockhus et al., 2014

#### Experts Review

After the preliminary preparation of the questionnaire, a total of eight persons, including experts in education, school teachers, teachers in after-school tutoring classes, psychological test experts, student parents, etc., are invited to jointly review all the items and provide suggestions on the topic description, meaning, and measurement scale. After the questions are fully revised based on their suggestions, the aforementioned experts are invited to conduct the second round of review. After confirming that no correction is required, the expert validity is established and the questionnaire is formally compiled.

### Analysis Approaches

**FIGURE 2:**
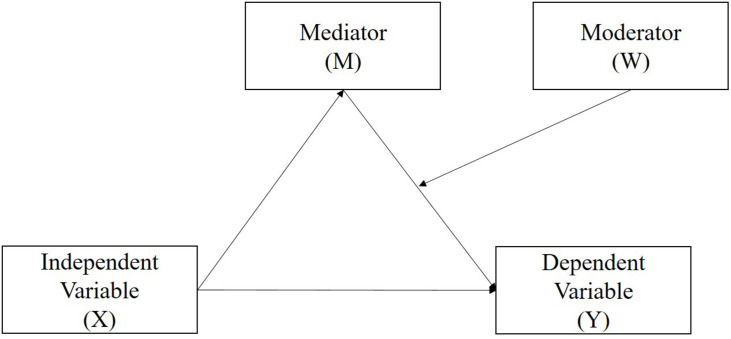
The conceptual framework of the second stage moderation of moderated mediation model.

Regression analysis is used to explain the strength of the variable relevant to the dependent variable to determine its predictive power. Its purpose is to explore the degree to which the response variable (Y) changes when the explanatory variable (X) changes; further, the moderator (W) will be moderating the relationships between X and Y (MacKinnon et al., 2002; Shrout and Bolger, 2002). To explore the causal relationships among parenting style, parent–child relationships, after-school program, and students’ creative self-efficacy, this study employed a “second stage moderation model” (Edwards and Lambert, 2007), model C (Preacher et al., 2007) of the moderated mediation model that provided a model No. 14 (Hayes, 2013, 2018), as shown in Figure 2, to deal with the specified causal relationship issues.

## Results

### The Demographic Variance of Participants

The sample distribution is shown in Table 2. In terms of gender, half of the participants (50%) are males (*n* = 275) and half are females (*n* = 275). In terms of grade level, 5th grade has the most participants (*n* = 169, 30.7%), followed by 6th grade (*n* = 76, 13.8%) and 3rd grade (*n* = 74, 13.5%). 8th grade (*n* = 21, 3.8%) and 9th grade (*n* = 21, 3.8%) are the lowest in number. The sequence of selections are “required by parents” (*n* = 369, 42.1%), “needing after-school tutoring” (*n* = 161, 18.4%), “thinking that after-school tutoring is interesting” (*n* = 158, 18.0%), and “being required by school” (*n* = 24, 2.7%). It is obvious that most of the after-school tutees are required by their parents, who are relatively passive while the others are voluntary participants.

**TABLE 2:** Descriptive summary of demographic variables (*n* = 550).

Demographic variables	Groups	*n*	Percentage
Gender	Female	275	50
	Male	275	50
K9 Grade	1	49	8.9
	2	44	8.0
	3	74	13.5
	4	67	12.2
	5	169	30.7
	6	76	13.8
	7	29	5.3
	8	21	3.8
	9	21	3.8
Reasons of participant the afterschool program (multi-selection)	I need the program	161	18.4
	My friends attended	99	11.3
	It is interesting	158	18.0
	Parents required	369	42.1
	As per teacher’s request	66	7.5
	School required	24	2.7

### Reliability and Validity Examination

#### Reliability Check

In order to confirm the reliability of the scale, the Cronbach’s α was employed to test the reliability (Cronbach and Shavelson, 2004; van der Ark et al., 2011; Dunn et al., 2014). The composite reliability (ρc) (Peterson and Kim, 2013; Padilla and Divers, 2016) and average variance extracted (AVE) (Costea et al., 2017) were also applied to test the reliability of the instrument. The results are shown in Table 3.

**TABLE 3:** The examined reliability coefficients summary (*n* = 550).

Factors	Items	Cronbach’s α	Composite reliability (ρc)	AVE
Parenting style – Emotional warmth (PS-EW)	4	0.766	0.845	0.578
Parenting style – punitive discipline (PS-PD)	4	0.821	0.784	0.551
Parenting style – anxious intrusiveness (PS-AI)	4	0.791	0.877	0.705
Parenting style – autonomy and support (PS-AS)	4	0.815	0.766	0.536
Positive parent – Child relationships (PCR+)	5	0.718	0.781	0.545
Negative parent – Child relationships (PCR-)	4	0.762	0.806	0.581
After-school program (ASP)	6	0.924	0.940	0.724
Creative self-efficacy (CSE)	12	0.935	0.944	0.585

The research questionnaire has a total of 40 evaluation variables, and the Cronbach’s α coefficients for each variable range from 0.594 to 0.935, all of which are greater than the acceptable standard value of more than 0.50 (Schweizer, 2011; Adamson and Prion, 2013). The values of combined reliability range from 0.781 to 0.944, which are higher than the standard value of more than 0.70 (Bagozzi and Yi, 1988; Peterson and Kim, 2013; Padilla and Divers, 2016). The average variation extraction (AVE) ranges from 0.551 to 0.724, which are higher than the standard value of 0.50 (Oye et al., 2014; Costea et al., 2017). Accordingly, the analysis results support the reliability of the questionnaire.

#### Constructive Validity Check

The convergent validity check was conducted to verify the factor loading should be grate than or equal to 4.0, and *T* statistics coefficient higher than 1.96 (Abazov et al., 2016; Black and Babin, 2019). The result shows in Table 4. The factor loading of 43 items were ranged from 0.666 to 0.883; the *T* statistics were all grate than 1.96 that reached the significant level.

**TABLE 4:** The examined convergent coefficients summary (*n* = 550).

Factors	Items	Factor loading	*T* Statistics (|O/STDEV|) > 1.96
Parenting style –	ParSty01A	0.766	26.569***
Emotional Warmth	ParSty02A	0.813	36.162***
	ParSty03A	0.751	21.801***
	ParSty04A	0.706	20.838***
Parenting style –	ParSty05B	0.813	25.340***
Punitive Discipline	ParSty06B	0.722	16.091***
	ParSty07B	0.728	15.710***
	ParSty08B	0.731	18.909***
Parenting style –	ParSty09C	0.860	41.330***
Anxious Intrusiveness	ParSty10C	0.828	33.616***
	ParSty11C	0.830	34.058***
	ParSty12C	0.798	29.873***
Parenting style –	ParSty13D	0.785	10.265***
Autonomy & Support	ParSty14D	0.819	9.290***
	ParSty15D	0.720	5.497***
	ParSty16D	0.708	5.124***
Positive	ParRel01A	0.666	11.009***
Parent-Child Relationships	ParRel02A	0.834	26.365***
	ParRel03A	0.816	32.562***
	ParRel04A	0.704	13.477***
	ParRel05A	0.736	19.203***
Negative	ParRel06B	0.820	25.327***
Parent-Child Relationships	ParRel07B	0.793	21.848***
	ParRel08B	0.777	25.688***
	ParRel09B	0.762	23.786***
After-school program	ASP01	0.831	36.432***
	ASP02	0.820	34.575***
	ASP03	0.872	47.989***
	ASP04	0.883	62.187***
	ASP05	0.842	46.339***
	ASP06	0.855	48.245***
Creative Self-efficacy	Creat01	0.721	26.240***
	Creat02	0.761	32.132***
	Creat03	0.771	33.576***
	Creat04	0.741	29.829***
	Creat05	0.772	32.337***
	Creat06	0.772	32.398***
	Creat07	0.737	26.669***
	Creat08	0.769	30.068***
	Creat09	0.754	24.902***
	Creat10	0.787	33.578***
	Creat11	0.832	50.947***
	Creat12	0.754	30.642***

***p < 0.001.

Finally, the analysis of discrimination validity adopts the HTMT (the heterotrait–monotrait ratio of correlations) method; that is, the confidence interval (C.I.) between facets and facets does not include 1.0 (Voorhees et al., 2016), as shown in Table 5. The results show that the C.I. coefficients range from 0.104 to 0.639, and none of them include 1.0, which supports the research scale to have discrimination validity.

**TABLE 5:** The divergent matrix coefficients summary (*n* = 550).

Factors	ASP	CSE	PCR-Positive	PCR-Negative	PS-EW	PS-PD	PS-AI
CSE	0.423						
PCR-Positive	0.189	0.318					
PCR-Negative	0.298	0.336	0.610				
PS-EW	0.104	0.160	0.135	0.228			
PS-PD	0.304	0.432	0.261	0.493	0.276		
PS-AI	0.230	0.261	0.206	0.177	0.215	0.116	
PS-AS	0.093	0.096	0.156	0.225	0.493	0.245	0.241

*ASP, After-school Program; CSE, Creative Self-Efficacy; PCR, Parent – Child Relationships; PS, Parenting Style; EW, Emotional Warmth; PD, Punitive Discipline; AI, Anxious Intrusiveness; AS, Autonomy and Support.*

### The Estimates of the Hypothetical Model

#### Parenting Style – Emotional Warmth

The “Parenting Style – Emotional Warmth” as an independent variable (X) to predict “Positive Parent–Child Relationships” (M_1_), “Negative Parent–Child Relationships” (M_2_), and the dependent variable “Creative Self-efficacy” (Y) then computed the coefficient of “After-school Program” (W) to Y and WM_1_, and WM_2_ to Y. Table 6 shows pathways of the model.

**TABLE 6:** The moderated mediation estimated coefficients summary: PS-EW (*n* = 550).

	Dependent Variables							
Independent Variables	Mediator (Parent–Child Relationships)				Y (Creative Self-Efficacy)			
		*b*	SE	*p*		*b*	SE	*p*
Constant	i_M_	−1.043	0.078	<0.001	i_Y_	3.392	0.241	<0.001
X_1 (PS–EW to PCR+)_	a_1_	**0.198**	**0.014**	**<0.001**	c’	**0.285**	**0.045**	**<0.001**
X_2(PS–EW to PCR–)_	a_2_	**−0.072**	**0.029**	**<0.050**		–	–	–
M_1 (PCR+)_		–	–	–	b_1_	0.122	0.111	>0.050
M_2 (PCR–)_		–	–	–	b_2_	−0.028	0.053	>0.050
W_(ASE)_		–	–	–	b_3_	**0.384**	**0.046**	**<0.001**
M_1 (PCR+)_ × W_(ASE)_		–	–	–	b_13_	−0.002	0.067	>0.050
M_2 (PCR–)_ × W_(ASE)_		–	–	–	b_23_	−0.015	0.046	>0.050
PCR+		PCR−						
*R*^2^ = 0.256		*R*^2^ = 0.011				*R*^2^ = 0.317		
*F*_(1, 548)_ = 188.657		*F*_(1, 548)_ = 6.105				*F*_(6, 543)_ = 42.091		
*p* < 0.001		*p* < 0.050				*p* < 0.050		

*The bold font was the path coefficients reached the significant level (p < 0.05). ASP, After-school Program; CSE, Creative Self-Efficacy; PCR, Parent–Child Relationships; PS, Parenting Style; EW, Emotional Warmth; PD, Punitive Discipline; AI, Anxious Intrusiveness; AS, Autonomy and Support.*

It reflected that the “Parenting Style – Emotional Warmth” can predict the “Positive Parent–Child Relationships” (X_1_) (β = 0.198, *p* < 0.001), “Negative Parent–Child Relationships” (X_2_) (β = −0.072, *p* < 0.05), and “Creative Self-efficacy” (Y) (β = 0.285, *p* < 0.001) significantly; however, the “Positive Parent–Child Relationships” (M_1_) (β = 0.122, *p* > 0.05) and “Negative Parent–Child Relationships” (M_2_) (β = −0.028, *p* > 0.05) did not associate with “Creative Self-efficacy” (Y). Further, the moderation effect did not exist in this model (WM_1_ and WM_2_ to Y).

#### Parenting Style – Punitive Discipline

**FIGURE 3:**
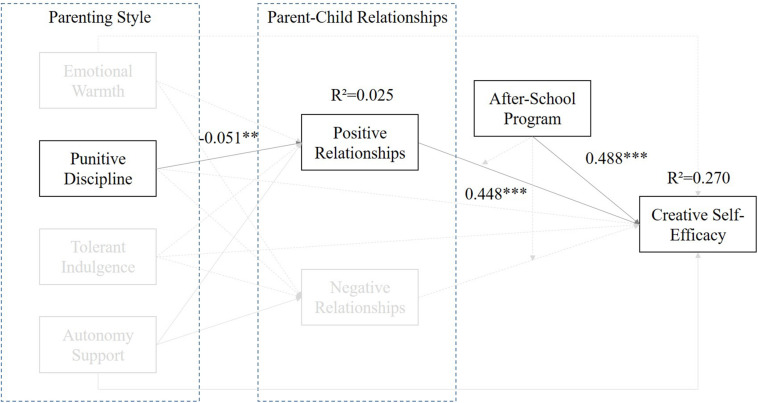
The standard coefficients of the prediction model of Parenting Style – Punitive Discipline.

Table 7 shows pathways of the model. It reflected that the “Parenting Style – Punitive Discipline” can predict the “Positive Parent–Child Relationships” (X_1_) (β = −0.051, *p* < 0.01) and the “Negative Parent–Child Relationships” (X_2_) (β = 0.174, *p* < 0.001) significantly; meanwhile, the “Positive Parent–Child Relationships” (M_1_) (β = 0.448, *p* < 0.001) was associated with the “Creative Self-efficacy” (Y), but the “Negative Parent–Child Relationships” (M_2_) (β = −0.076, *p* > 0.05) was not. Further, the moderation effect (WM_1_ and WM_2_ to Y) did not exist in this model, but the “After-school Program” (W) (β = −0.488, *p* < 0.001) can predict Y significantly as well. The result is shown in Figure 3.

**TABLE 7:** The moderated mediation estimated coefficients summary: PS-PD (*n* = 550).

	Dependent Variables							
Independent Variables	Mediator (Parent–Child Relationships)				Y (Creative Self-Efficacy)			
		*b*	SE	*p*		*b*	SE	*p*
Constant	i_M_	0.176	0.052	<0.010	i_Y_	3.392	0.241	<0.001
X_1 (PS–PD to PCR+)_	a_1_	**−0.051**	**0.014**	**<0.010**	c’	0.049	0.032	>0.050
X_2(PS–PD to PCR–)_	a_2_	**0.174**	**0.023**	**<0.001**		–	–	–
M_1 (PCR+)_		–	–	–	b_1_	**0.448**	**0.106**	**<0.001**
M_2 (PCR–)_		–	–	–	b_2_	−0.076	0.057	>0.050
W_(ASE)_		–	–	–	b_3_	**0.488**	**0.044**	**<0.001**
M_1 (PCR+)_ × W_(ASE)_		–	–	–	b_13_	0.029	0.069	>0.050
M_2 (PCR–)_ × W_(ASE)_		–	–	–	b_23_	0.070	0.048	>0.050
PCR+		PCR−						
*R*^2^ = 0.025		*R*^2^ = 0.098				*R*^2^ = 0.270		
*F*_(1, 548)_ = 14.191		*F*_(1, 548)_ = 59.170				*F*_(6, 543)_ = 33.410		
*p* < 0.010		*p* < 0.010				*p* < 0.001		

*The bold font was the path coefficients reached the significant level (p < 0.05). ASP, After-school Program; CSE, Creative Self-Efficacy; PCR, Parent–Child Relationships; PS, Parenting Style; EW, Emotional Warmth; PD, Punitive Discipline; AI, Anxious Intrusiveness; AS, Autonomy and Support.*

#### Parenting Style – Anxious Intrusiveness

Table 8 shows pathways of the model. It reflected that the “Parenting Style – Anxious Intrusiveness” can predict the “Negative Parent–Child Relationships” (X_2_) (β = 0.100, *p* < 0.01) and “Creative Self-efficacy” (Y) (β = 0.099, *p* < 0.05) significantly but did not associate with the “Positive Parent–Child Relationships” (X_1_) (β = −0.18, *p* > 0.05). Further, the “Positive Parent–Child Relationships” (M_1_) (β = 0.419, *p* < 0.001) was associated with the “Creative Self-efficacy” (Y), but the “Negative Parent–Child Relationships” (M_2_) (β = −0.077, *p* > 0.05) did not associate with Y. Further, the moderation effect (WM_1_ and WM_2_ to Y) did not exist in this model, but the “After-school Program” (W) (β = −0.478, *p* < 0.001) can predict Y significantly.

**TABLE 8:** The moderated mediation estimated coefficients summary: PS-AI (*n* = 550).

	Dependent Variables							
Independent Variables	Mediator (Parent–Child Relationships)				Y (Creative Self-Efficacy)			
		*b*	SE	*p*		*b*	SE	*p*
Constant	i_M_	−0.054	0.049	>0.050	i_Y_	4.601	0.111	<0.001
X_1 (PS–AI to PCR+)_	a_1_	0.018	0.015	>0.050	c’	**0.099**	**0.033**	**<0.050**
X_2(PS–AI to PCR–)_	a_2_	**0.100**	**0.025**	**<0.010**		–	–	–
M_1 (PCR+)_		–	–	–	b_1_	**0.419**	**0.104**	**<0.010**
M_2 (PCR–)_		–	–	–	b_2_	−0.077	0.055	>0.050
W_(ASE)_		–	–	–	b_3_	**0.478**	**0.044**	**<0.001**
M_1 (PCR+)_ × W_(ASE)_		–	–	–	b_13_	0.035	0.068	>0.050
M_2 (PCR–)_ × W_(ASE)_		–	–	–	b_23_	0.001	0.048	>0.050
PCR+		PCR−						
*R*^2^ = 0.003		*R*^2^ = 0.028				*R*^2^ = 0.528		
*F*_(1, 548)_ = 1.533		*F*_(1, 548)_ = 15.695				*F*_(6, 543)_ = 34.899		
*p* > 0.050		*p* < 0.010				*p* < 0.001		

*The bold font was the path coefficients reached the significant level (p < 0.05). ASP, After-school Program; CSE, Creative Self-Efficacy; PCR, Parent-Child Relationships; PS, Parenting Style; EW, Emotional Warmth; PD, Punitive Discipline; AI, Anxious Intrusiveness; AS, Autonomy and Support.*

#### Parenting Style – Autonomy and Support

**FIGURE 4:**
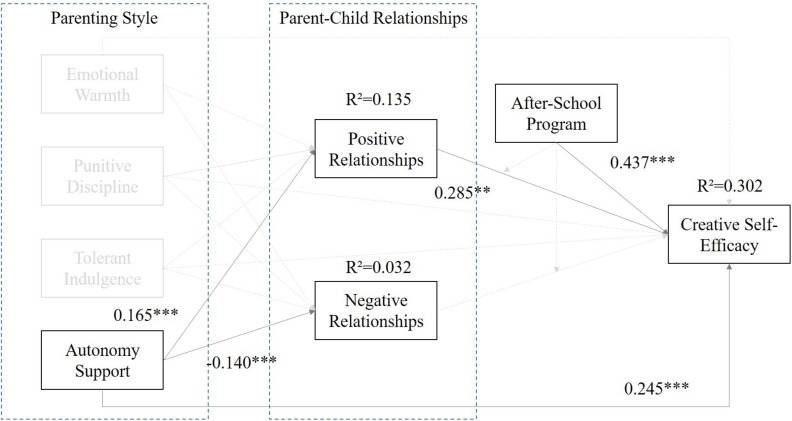
The standard coefficients of the prediction model of Parenting Style – Autonomy and Support.

Table 9 shows pathways of the model. It reflected that the “Parenting Style – Punitive Discipline” can predict the “Positive Parent–Child Relationships” (X_1_) (β = −0.165, *p* < 0.001), “Negative Parent–Child Relationships” (X_2_) (β = −0.140, *p* < 0.001), and “Creative Self-efficacy” (Y) (β = 0.245, *p* < 0.001) significantly. Meanwhile, the “Positive Parent–Child Relationships” (M_1_) (β = 0.285, *p* < 0.001) was associated with the “Creative Self-efficacy” (Y), but the “Negative Parent–Child Relationships” (M_2_) (β = −0.005, *p* > 0.05) did not associate with Y. Further, the moderation effect (WM_1_ and WM_2_ to Y) did not exist in this model, but the “After-school Program” (W) (β = −0.437, *p* < 0.001) can predict Y significantly. The result is shown in Figure 4.

**TABLE 9:** The moderated mediation estimated coefficients summary: PS-AS (*n* = 550).

	Dependent Variables							
Independent Variables	Mediator (Parent–Child Relationships)				Y (Creative Self-Efficacy)			
		*b*	SE	*p*		*b*	SE	*p*
Constant	i_M_	−0.989	0.109	<0.001	i_Y_	3.421	0.287	<0.001
X_1 (PS–AS to PCR+)_	a_1_	**0.165**	**0.018**	**<0.001**	c’	**0.245**	**0.047**	**<0.001**
X_2(PS–AS to PCR–)_	a_2_	**–0.140**	**0.033**	**<0.001**		–	–	–
M_1 (PCR+)_		–	–	–	b_1_	**0.285**	**0.106**	**<0.010**
M_2 (PCR–)_		–	–	–	b_2_	−0.005	0.0541	>0.050
W_(ASE)_		–	–	–	b_3_	**0.437**	**0.044**	**<0.001**
M_1 (PCR+)_ × W_(ASE)_		–	–	–	b_13_	0.054	0.067	>0.050
M_2 (PCR–)_ × W_(ASE)_		–	–	–	b_23_	−0.007	0.047	>0.050
PCR+		PCR-						
*R*^2^ = 0.135		*R*^2^ = 0.032				*R*^2^ = 0.302		
*F*_(1, 548)_ = 85.406		*F*_(1, 548)_ = 17.921				*F*_(6, 543)_ = 39.068		
*p* < 0.001		*p* < 0.001				*p* < 0.001		

*The bold font was the path coefficients reached the significant level (p < 0.05). ASP, After-school Program; CSE, Creative Self-Efficacy; PCR, Parent–Child Relationships; PS, Parenting Style; EW, Emotional Warmth; PD, Punitive Discipline; AI, Anxious Intrusiveness; AS, Autonomy and Support.*

## Conclusion

### Cultivate Parent’s Attitude Toward Parenting

From the perspective of family support systems, parenting styles, and family atmosphere, parents’ attitudes toward children are the key to whether students’ creativity can be inspired. If parents believe in a democratic way of communication and are willing to treat their children by respecting individual differences, such a communication environment is sure to be a favorable environment and used the information and communication technology for the fostering of children’s creativity (Reppa et al., 2010; Yuan and Wu, 2020). Based on the analysis results, only punitive discipline and autonomy support of the parenting style will impact positive relationships significantly. Further, positive parent–child relationships and the after-school program can predict students’ self-efficacy positively. Those results demonstrated only positive parent–child relationships. The path “punitive discipline–positive parent–child relationships–creative self-efficacy” (see Figure 3) supported a complete mediation effect among those three factors; in particular, the punitive discipline will negatively impact the relationships of parent and child. It reflected that the parents with punitive discipline referenced the parenting style of an Eastern society due to the highly competitive environment and cultural context (Chen and Hsu, 2011). Moreover, Eastern parents usually have high expectations for their children in that parents will push their children to study harder to get a higher academic achievement and competency strength, causing tension between parents and children (Gu et al., 2017). However, positive parent–child relationships will affect their children’s creative self-efficacy directly. Zhang et al. (2018) found that children’s creativity will be inspired by parent–child relationships directly. This study’s findings matched Zhang et al.’s result and supported the idea that positive parent–child relationships can construct a good environment to cultivate children’s creative self-efficacy. It is important for children to establish their foundations of competency and creativity. These results reminded parents to not put too much stress on children for it will harm their relationships negatively. In addition, maintaining positive parent–child relationships can promote children’s creative self-efficacy significantly, and it is helpful for children’s creativity and competency.

### The Positive Parent–Child Relationships and Autonomy Support From Parent

From the results of the above analysis, it is found that the parent–child relationships will be affected by autonomy support parenting style largely. The path “autonomy support–positive parent–child relationships–creative self-efficacy” (see Figure 4) supported partial mediation effects among those three factors. Following the globalization and ICT (information and communication technology) development in the recent decades, it is easy to find the significant difference between new generation of Eastern young parents and traditional parents. The young parents are more open-minded and flexibility on autonomy for children. These results show that the Eastern parenting style is more close to Western culture (Garg et al., 2005). Compared to the punitive discipline parenting style, if the parents adopt the style of authoritative or paternalistic leadership in the upbringing of their children when they are young, then the children will be pressured to always make the correct choice when they are authorized to make their own decisions. Furthermore, the autonomy support parenting style will affect students’ creative self-efficacy directly. It shows that the student’s creative self-efficacy will be affected not only by positive parent–child relationships but also by autonomy support parenting style. This result argued that parents can inspire or cultivate their children’s creative self-efficacy by autonomy support parenting style and positive parent–child relationships (Zhang et al., 2018). Thus, it is recommended that parents should treat their children via autonomy style to enhance children’s self-regulation and judgment and then give them adequate support to assist them to overcome barriers or equip them with problem-solving ability.

### The Contribution of After-School Programs

Based on the study results, no moderation effects appeared in the research model. It shows that the after-school program was separated from parent–child relationships that affected students’ creative self-efficacy, respectively. Thus, the after-school program will affect students’ creative self-efficacy independently and it promoted the value of the after-school program. Moreover, as after-school tutoring has a significant positive influence on students ‘creative self-efficacy, the in-depth analysis found that this might be so due to the fact that the after-school tutoring classes are more relaxed and give students more autonomy support than they can enjoy at home. In addition, counseling courses or skill learning courses in after-class tutoring programs have more activities designed to encourage students to make their own decisions or to lead, which often play an important role in inspiring students’ creativity. Besides, students in the after-school counseling classes usually join the programs out of their own will. They are more active, which will in turn cause the counseling courses to have a direct and positive impact on students’ creative self-efficacy.

In comparison with the previous literature, the results of this study provide more evidence to support the view that after-school tutoring courses do have a great impact on students’ creative self-efficacy. It also fills the gaps in the academic research concerning how after-school tutoring might impact students’ creative self-efficacy, especially in the context of the Chinese society where social values are usually suppressed in the family. After-school counseling places children in an environment of less authoritative leadership, which is sure to be more helpful to inspire creativity and improve their creative self-efficacy.

Based on the above discussion, this study puts forward relevant research suggestions for future researchers’ reference: (1) Considering the differences between the physiological and psychological maturity stages, the age sampling of research can focus more on grouping. It is recommended that the sampling can be divided into early grades (grades 1 to 3 of elementary school), middle grades (4th grade to 6th grade in elementary school), senior grades (7th grade to 9th grade in middle school), and so on. (2) The research objects can be expanded to compare programs across countries (at home and abroad), or, more specifically, to adopt qualitative interview method in order to look deeper into the differences between Chinese and Western cultures in terms of upbringing methods and upbringing concepts. (3) It is recommended to add research tools and research variables in a timely manner. As theories are often affected by social changes, there may be more variables in the parenting style and parent–child relationship that should be taken into consideration so as to maintain the reliability and validity of the research results. (4) It is recommended to adjust the research model that might better explain updated data. Due to the fact that the causal relationships among parenting styles, parent–child relationships, after-school counseling, and creative self-efficacy are still changing dynamically with different crowds, it is therefore necessary to revise the research model in a timely fashion so as to clarify and present the most realistic influences among variables.

### Strengths and Limitations

The strengths of this study are advanced and ahead of the trend of after-school and creativity cultivation-related studies. Further, this study constructed a reliable instrument to measure the proposed factors, and the results are meaningful valuable contributions to informal education studies. Moreover, in accordance with research findings, the parents should review their parenting style and the relationships with their children carefully to find new ways to deal with their expectations and education problem, especially in the Chinese cultural context.

The limitations of this study are as follows: (1) The age range (from 9 to 16) of the participants was compared largely; it will affect the results of the analysis due to cognition and awareness being different between samples of different ages. (2) The samples were selected from an Asian society with a Chinese cultural context; thus, the results are not suitable for global comparison because the interaction between parents and children in the study is different from Western society.

## Data Availability Statement

The raw data supporting the conclusions of this article will be made available by the authors, without undue reservation, to any qualified researcher.

## Ethics Statement

The studies involving human participants were reviewed and approved by the Yuanpei University of Medical Technology of Human Research Ethics Committee (HREC, Hsinchu, Taiwan, https://hrec.ypu.edu.tw/). The participants and/or their parents provided written informed consent to participate in this study.

## Author Contributions

C-CL planned the project and she has done the questionnaire survey, data collection, and selection. Y-HY generated the survey items, the validity, reliability check, statistical analysis, and the report written. Both authors contributed to the article and approved the submitted version.

## Conflict of Interest

The authors declare that the research was conducted in the absence of any commercial or financial relationships that could be construed as a potential conflict of interest.
